# Spatial factors for COVID-19 associated community deaths in an urban area of Lusaka, Zambia: an observational study

**DOI:** 10.11604/pamj.2023.45.32.37069

**Published:** 2023-05-15

**Authors:** Amos Hamukale, Tadatsugu Imamura, Muzala Kapina, Olena Borkovska, Chisenga Abel Musuka, Emmanuel Tembo, Yingtao Xie, Carmen Tedesco, Paul Msanzya Zulu, Patrick Sakubita, Fred Kapaya, Raymond Hamoonga, Mazyanga Lucy Mazaba, Chie Nagata, Akira Ishiguro, Nathan Kapata, Victor Mukonka, Nyambe Sinyange

**Affiliations:** 1 Public Health National Tuberculosis and Leprosy Program, Ministry of Health, Lusaka, Zambia,; 2 Japan International Cooperation Agency, Tokyo, Japan,; 3 Center for Postgraduate Education and Training, National Center for Child Health and Development, Tokyo, Japan,; 4 National Public Health Institute, Lusaka, Zambia,; 5 Geo-Referenced Infrastructure and Demographic Data for Development, Columbia University, New York, USA,; 6 Zambia Data Hub, National Spatial Data Infrastructure (NSDI), Lusaka, Zambia,; 7 Ministry of Lands and Natural Resources, Lusaka, Zambia,; 8 Department of Analytics, Fraym Arlington, Virginia, USA

**Keywords:** COVID-19, spatial epidemiology, community death, unplanned residential area, education level, Zambia

## Abstract

We retrospectively analyzed spatial factors for coronavirus disease 2019 (COVID-19)-associated community deaths i.e., brought-in-dead (BID) in Lusaka, Zambia, between March and July 2020. A total of 127 cases of BID with geocoordinate data of their houses were identified during the study period. Median interquartile range (IQR) of the age of these cases was 49 (34-70) years old, and 47 cases (37.0%) were elderly individuals over 60 years old. Seventy-five cases (75%) of BID were identified in July 2020, when the total number of cases and deaths was largest in Zambia. Among those whose information regarding their underlying medical condition was available, hypertension was most common (22.9%, 8/35). Among Lusaka’s 94 townships, the numbers (median, IQR) of cases were significantly larger in those characterized as unplanned residential areas compared to planned areas (1.0, 0.0-4.0 vs 0.0, 0.0-1.0; p=0.030). The proportion of individuals who require more than 30 minutes to obtain water was correlated with a larger number of BID cases per 105 population in each township (rho=0.28, p=0.006). The number of BID cases was larger in unplanned residential areas, which highlighted the importance of targeted public health interventions specifically to those areas to reduce the total number of COVID-19 associated community deaths in Lusaka. Brought-in-dead surveillance might be beneficial in monitoring epidemic conditions of COVID-19 in such high-risk areas. Furthermore, inadequate access to water, sanitation, and hygiene (WASH) might be associated with such distinct geographical distributions of COVID-19 associated community deaths in Lusaka, Zambia.

## Introduction

Coronavirus disease 2019 (COVID-19) mortality rate in Africa has been thought to be lower than in other parts of the world [1-3]. However, there are discussions over the true burden of COVID-19 mortality in low- and middle-income countries (LMICs), including sub-Saharan Africa, as some researchers raised a possibility of underestimated COVID-19 mortality due to limited testing capacities, poor registration systems, and uncaptured fatal cases in the communities [4]. Despite the increasing significance of defining the public health impact of COVID-19-associated deaths in the communities of LMICs, only very limited information is available on the occurrence of COVID-19 associated community deaths and geospatial factors associated with such events, particularly in sub-Saharan African countries. In this study, we aim to determine the spatial factors for COVID-19 associated community deaths in an urban area of Lusaka, Zambia, by retrospectively reviewing postmortem surveillance data collected between March and July 2020.

## Methods

**Figure 1:**
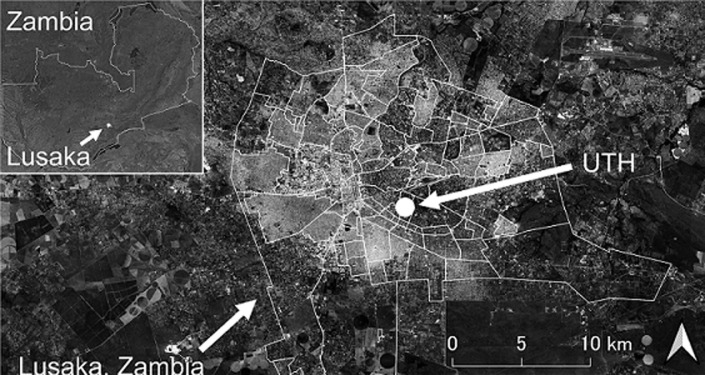
map of Lusaka, Zambia

The study was conducted in Lusaka, the capital city of Zambia (Figure 1). The city consists of planned and unplanned residential areas. Planned residential areas are those officially created by the Government Planning Authority whereas unplanned residential areas are not. We conducted a retrospective data analysis of COVID-19 cases that were identified in postmortem surveillance for brought-in-dead (BID) cases (BID surveillance) between March 18 (the date of first COVID-19 case identification in Zambia) and July 31, 2020, in Lusaka, Zambia. In the BID surveillance, nasopharyngeal swabs were collected from individuals who were admitted to the University Teaching Hospital (UTH) mortuary, Lusaka, after having died in the community. These respiratory samples were subjected to COVID-19 testing (i.e., polymerase chain reaction or point-of-care antigen testing) at UTH. Patient information and geocoordinate data for houses of BID cases were collected by physical visits to these houses once informed consent was given by family members or guardians of the cases.

Ethics approval for the use of patients´ data collected as part of the public health response of Zambia National Public Health Institute and the Ministry of Health, Zambia for analysis and publication was obtained from the Zambia National Health Research Authority (reference number NHRA00002/31/03/2022). Digital maps of Lusaka showing the geographical distribution of BID cases and associated factors were developed using QGIS version 3.10 A Coruña. Township boundaries were accessed through the Zambia Data Hub where they were provided for download by the Ministry of Lands, the Office of Surveyor General. The geographical distribution of associated factors was developed by Fraym as part of the Geo-Referenced Infrastructure and Demographic Data for Development (GRID3) project (Table 1) [5]. The numbers of BID cases and proportions of associated factors were calculated for each of these 94 townships using QGIS.

**Table 1:** definitions of variables produced using the data from the demographic Health Survey (DHS) 2018

Variables	Definition
Proportion of male gender	Number of males divided by total number of residents in the area
Proportion of elderly persons ≥ 60 years old	Number of elderly persons (≥ 60 years old) divided by total number of residents in the area
Population density	Number of residents divided by the area size (km^2^)
Proportion of radio listeners	Number of adults (15-49 years old) that listen to radio >= once per week divided by the number of adult residents in the area
Proportion of individuals without access to internet	Number of residents in a household without internet divided by total number of residents in the area
Proportion of individuals without televisions at home	Number of residents in a household without televisions divided by total number of residents in the area
Proportion of adults employed in essential occupations	Number of adults employed in essential occupations (e.g., armed forces, health professionals) divided by total number of adult residents in the area
Proportion of individuals without education higher than primary level	Number of residents (≥15 years old) who completed primary education (grade 1-7 between 7-13 years old), but do not have any secondary or higher education divided by total number of residents aged 15 years and above in the area
Proportion of individuals without soap/detergent at home	Number of residents who do not have any soap or detergent at home divided by total number of residents in the area
Proportion of individuals without piped-in drinking water at home	Number of residents who do not have piped-in drinking water at home divided by total number of residents in the area
Proportion of individuals without water for hand washing at home	Number of residents who do not have water for hand washing at home divided by total number of residents in the area
Proportion of individuals who require more than 30 minutes to obtain water	Number of residents who require more than 30 minutes to obtain water divided by total number of residents in the area
Proportion of individuals who share toilets with others or do not have toilets at home	Number of residents who share toilets with other houses or those who do not have toilets at home divided by total number of residents in the area

Statistical analysis was conducted using R ver.3.5.0 (R Foundation for Statistical Computing, Vienna, Austria). We performed a Wilcoxon rank sum test to compare numbers of BID cases and numbers of BID cases per 105 population between the different types of townships. The Spearman´s rank correlation coefficient was used to calculate the correlation between numbers of BID cases per 105 population and spatial factors in continuous variables. Rho being equal to or greater than 0.2 was regarded as a positive correlation [6]. A p-value less than 0.05 was considered as statistically significant.

## Results

**Figure 2:**
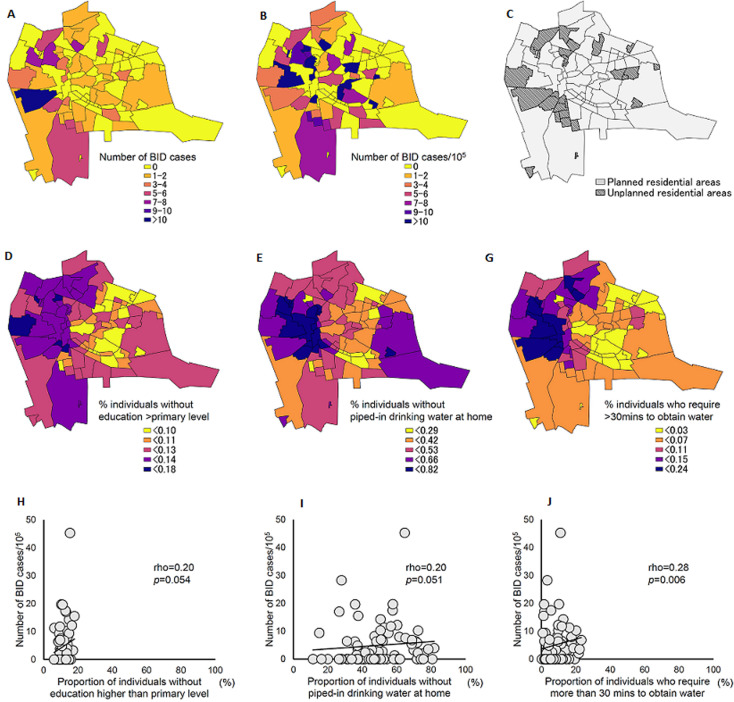
geographical distribution of COVID-19 positive brought-in-dead cases and spatial factors in Lusaka, Zambia, between March 18 and July 31, 2020

A total of 189 COVID-19 positive BID cases were identified in Lusaka, March-July 2020. Among them, geocoordinate data of their houses were available for 127 cases (67.2%). Ninety-five cases (75%) were identified in July 2020. Median interquartile range (IQR) of the age of these cases was 49 (34-70) years old. Information regarding underlying medical conditions was available for 35 cases; hypertension (22.9%, 8/35) was the most common underlying medical condition, which was followed by diabetes and cancer (20.0%, 7/35) (Table 2). Information about clinical symptoms prior to admission was available for only 29 cases (22.8%, 29/127), among which fever and stomach ache were the most common symptoms (17.2%, 5/29) (Table 2). Out of 94 townships in Lusaka, COVID-19 positive BID cases were identified in 42 townships (45%, 42/94) (Figure 2). The number (median, IQR) of BID cases was larger in unplanned residential areas than in planned residential areas (1.0, 0.0-4.0 vs 0.0, 0.0-1.0; p=0.030). However, the number (median, IQR) of BID cases per 105 population was not significantly different between planned and unplanned residential areas (2.6, 0.0-6.7 vs 0.0, 0.0-6.3; p=0.574). A total of thirteen variables were explored for finding any correlations with the number of BID cases per 105 population in each township (Figure 2, Table 3). The number of BID cases per 105 population had a significant correlation with the proportion of individuals who required more than 30 minutes to obtain water (rho=0.28, p=0.006) (Figure 2). The proportion of individuals without education higher than primary level, and those without piped-in drinking water at home, showed a positive correlation with the number of BID cases per 105 population, although they did not reach statistical significance (rho=0.20, p=0.054 and rho=0.20, p=0.051, respectively) (Figure 2).

**Table 2:** characteristics of brought-in-dead cases with COVID-19 in Lusaka, Zambia, between March 18 and July 31, 2020

		Brought-in-dead cases with COVID-19 (n=127)
**Gender, female (%)**		74 (58.3%)
**Age, median (IQR)**		49 (34-70)
	> 60 years old	47 (37.0%)
**Residential area**		
	Planned	52 (40.9%)
	Unplanned	75 (59.1%)
Underlying medical conditions*		
	Hypertension	8 (22.9%)
	Diabetes	7 (20.0%)
	Cancer	7 (20.0%)
	Tuberculosis	5 (14.3%)
	HIV	2 (5.7%)
	Heart conditions	2 (5.7%)
	COPD	1 (2.9%)
Symptoms prior to admission*		
	Fever	5 (17.2%)
	Cough	4 (13.8%)
	Sore throat	2 (6.9%)
	Difficulty breathing	3 (10.3%)
	Chest pain	4 (13.8%)
	Vomiting	4 (13.8%)
	Stomachache	5 (17.2%)
	Diarrhea	2 (6.9%)

COPD: chronic obstructive pulmonary disease; HIV: Human immunodeficiency virus; IQR: interquartile range; BID: brought-in-dead

**Table 3:** correlation between spatial factors and numbers of COVID-19 positive brought-in-dead (BID) cases per 105 population in Lusaka, Zambia, between March 18 and July 31, 2020

Variables	Spearman’s rank correlation coefficient	p-value
Proportion of male gender	0.137	0.189
Proportion of elderly persons > 60 years old	-0.192	0.063
Population density	0.062	0.550
Proportion of radio listeners	-0.047	0.650
Proportion of individuals without access to internet	0.076	0.467
Proportion of individuals without televisions at home	-0.010	0.920
Proportion of adults employed in essential occupations	0.069	0.508
Proportion of individuals without education higher than primary level	0.201	0.054
Proportion of individuals without soap/detergent at home	0.066	0.527
Proportion of individuals without piped-in drinking water at home	0.202	0.051
Proportion of individuals who require more than 30 minutes to obtain water	0.279	0.006
Proportion of individuals who share toilets with others or do not have toilets at home	0.191	0.065

## Discussion

We reported the geographical distribution of COVID-19 positive BID cases and spatial factors associated with these BID cases in Lusaka, Zambia. This is one of the few studies describing the public health burden of COVID-19 associated community deaths and spatial factors for it in sub-Saharan African countries. In this study, the number of BID cases was larger in unplanned residential areas than in planned areas, potentially due to the large population size in unplanned areas. Our results highlighted the importance of targeted public health interventions specifically to those areas to reduce the total number of COVID-19-associated community deaths in an urban area of Lusaka, Zambia. Furthermore, a smaller prevalence of COVID-19 cases in unplanned residential areas was observed in our previous study, which was suggestive of under-reporting of cases in those areas (data will be presented elsewhere). In the present study, the number of BID cases was largest in July 2020, which was in parallel with the total number of COVID-19 cases and deaths in Zambia that reached its first peak in the same month [7]. Brought-in-dead surveillance might be beneficial in monitoring epidemic conditions of COVID-19 in such areas. Approximately 20% of BID cases reported underlying medical conditions that were known risk factors for severe clinical outcomes of COVID-19 [8]. The small proportion of elderly individuals over 60 years old might have been due to the generally young population in Zambia [9]. Notably, factors for inadequate access to water, sanitation, and hygiene (WASH) were associated with the increased number of BID cases per 105 population. Our results are suggestive of the importance of WASH to suppress COVID-19-associated mortality in Lusaka. Meanwhile, a potential correlation between inadequate access to WASH and other social factors (i.e., increased chances to contact neighbors for water and other daily necessities, low social economic status associated with reduced health-seeking behaviors) requires further evaluation. In addition, the proportion of individuals without education higher than the primary level was positively associated with the number of BID cases per 105 population, although it did not reach statistical significance. Comprehension of the risk communications delivered by health authorities might have been low due to the low education level, which could have led to reduced adherence to infection prevention measures and proper health-seeking behaviors. Improved risk communication strategies are warranted to deliver key messages to the high-risk population. Potential limitations of our study include the ecological study design using a relatively small number of BID cases, and limited information about the direct cause of death, adherence to mitigation measures, and economic activities of residents. Effects of postmortem testing on the sensitivity of identifying COVID-19 associated BID cases were assumed to be minimal, as we subjected specimens to testing immediately after admission of the cases [10].

## Conclusion

We observed a larger number of BID cases with COVID-19 in unplanned residential areas. Inadequate access to WASH was suggested as one of the factors associated with such distinct geographical distributions. Further studies are required to fully define the risk factors for COVID-19-associated community deaths and to establish efficient measures to suppress COVID-19 mortality in LMICs, including sub-Saharan African countries.

### What is known about this topic

- *The public health burden of COVID-19 mortality in low- and middle-income countries has not been fully defined, as there is a possibility of uncaptured fatal cases in the communities;*
- *Information on the occurrence of COVID-19 associated community deaths and geospatial factors associated with such events is limited*.

### What this study adds

- *The number of COVID-19 associated community deaths was larger in unplanned residential areas than in planned areas in Lusaka, Zambia;*
- *Factors for inadequate access to water, sanitation, and hygiene were associated with the increased number of COVID-19 associated community deaths in Lusaka, Zambia;*
- *Targeted public health interventions to high-risk areas (e.g., unplanned residential areas) might be important to reduce the total number of COVID-19 associated community deaths*.
